# Fatal Sepsis Associated with Bacterial Contamination of Platelets — Utah and California, August 2017

**DOI:** 10.15585/mmwr.mm6725a4

**Published:** 2018-06-29

**Authors:** Roberta Z. Horth, Jefferson M. Jones, Janice J. Kim, Bert K. Lopansri, Sarah J. Ilstrup, Joy Fridey, Walter E. Kelley, Susan L. Stramer, Ashok Nambiar, Lynn Ramirez-Avila, Amy Nichols, Wendy Garcia, Kelly F. Oakeson, Nicholas Vlachos, Gillian McAllister, Robert Hunter, Allyn K. Nakashima, Sridhar V. Basavaraju

**Affiliations:** ^1^Epidemic Intelligence Service, CDC; ^2^Division of Scientific Education and Professional Development, CDC; ^3^Utah Department of Health; ^4^Division of Healthcare Quality Promotion, National Center for Emerging and Zoonotic Infectious Disease, CDC; ^5^California Department of Public Health, Richmond, California; ^6^Intermountain Healthcare, Murray, Utah; ^7^American Red Cross Blood Services, Salt Lake City, Utah; ^8^American Red Cross Blood Services, Pomona, California; ^9^Scientific Office, American Red Cross, Gaithersburg, Maryland; ^10^University of California San Francisco; ^11^Davis County Health Department, Clearfield, Utah; ^12^Utah Public Health Laboratory, Utah Department of Health, Taylorsville, Utah.

During August 2017, two separate clusters of platelet transfusion–associated bacterial sepsis were reported in Utah and California. In Utah, two patients died after platelet transfusions from the same donation. *Clostridium perfringens* isolates from one patient’s blood, the other patient’s platelet bag, and donor skin swabs were highly related by whole genome sequencing (WGS). In California, one patient died after platelet transfusion; *Klebsiella pneumoniae* isolates from the patient’s blood and platelet bag residuals and a nontransfused platelet unit were matched using WGS. Investigation revealed no deviations in blood supplier or hospital procedures. Findings in this report highlight that even when following current procedures, the risk for transfusion-related infection and fatality persists, making additional interventions necessary. Clinicians need to be vigilant in monitoring for platelet-transmitted bacterial infections and report adverse reactions to blood suppliers and hemovigilance systems. Blood suppliers and hospitals could consider additional evidence-based bacterial contamination risk mitigation strategies, including pathogen inactivation, rapid detection devices, and modified screening of bacterial culture protocols

## Investigation and Results

**Utah cluster.** In August 2017, two apheresis platelet units and one unit of plasma were manufactured from an apheresis blood donation in Utah. Both platelet units were distributed to hospital X (Figure), where a male (patient A) with acute myeloid leukemia and neutropenia received one of the platelet units. Thirty minutes after transfusion, he developed rigors; transfusion-transmitted bacterial infection was not considered then because of the patient’s complex medical history. The patient died 4 days later. Anaerobic blood cultures, obtained shortly after transfusion, grew *C. perfringens* 5 days after collection.

**FIGURE:**
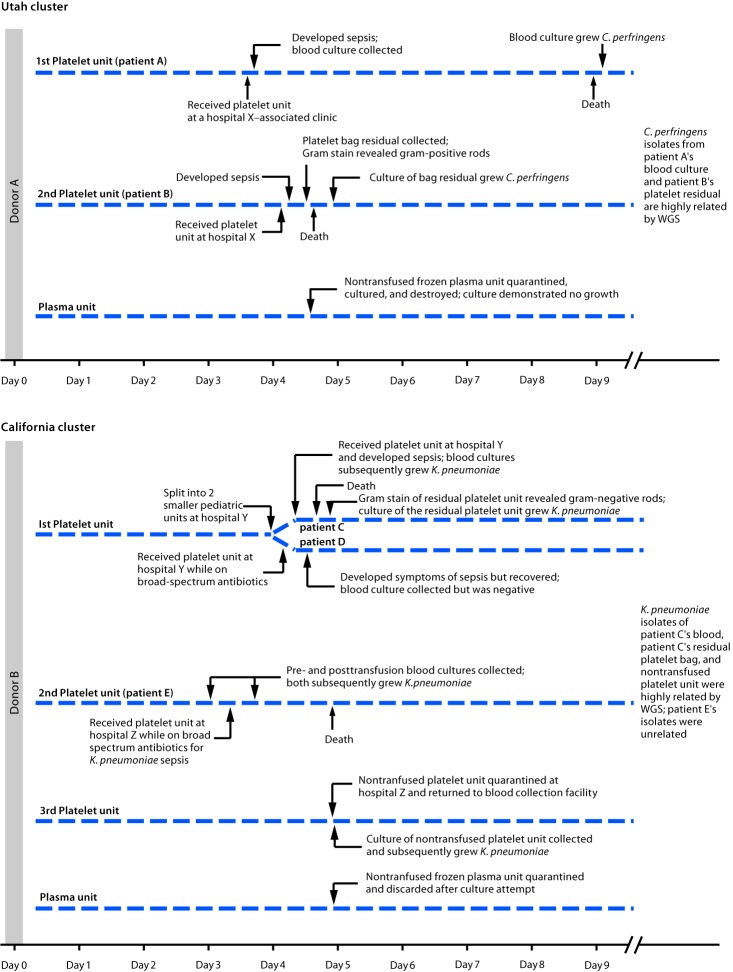
Timeline of two clusters of sepsis caused by bacterial contamination of platelets — Utah and California, August 2017 **Abbreviations:** *C. perfringens* = *Clostridium perfringens*; *K. pneumoniae* = *Klebsiella pneumoniae*; WGS = whole genome sequencing.

Fourteen hours after patient A’s transfusion, a female (patient B) with acute myeloid leukemia received the other platelet unit while on broad-spectrum antibiotics for neutropenia at hospital X. No immediate symptoms of sepsis followed transfusion. Later that day, routine laboratory testing revealed new intravascular hemolysis. Transfusion-transmitted bacterial infection was suspected, and Gram stain of platelet bag residuals was performed, revealing gram-positive bacilli; the platelet supplier was immediately notified. Patient B died 11 hours after transfusion. *C. perfringens* was isolated from an anaerobic culture of the residual platelets. Posttransfusion blood cultures from patient B were negative.

Platelet units transfused to patients A and B had been collected 4 days before transfusion (Figure). Routine inoculation for aerobic culture, performed 24 hours after donation, was negative for bacterial growth through 5 days.

The donor had previously donated platelets and whole blood with no recipient adverse reactions reported. The health department interviewed the donor, who reported no relevant infectious exposures or symptoms. The donor consented to skin swabs, collected from the axillae, antecubital fossae, and anus. Consent for environmental sampling was not provided by the donor.

As part of the investigation, multiple samples from the donor, recipients, and platelet bags were cultured for *C. perfringens* under anaerobic conditions. DNA was isolated from cultures that had growth (donor axillae and both antecubital fossae swabs, patient A’s blood, two isolates of patient B’s platelet bag residual, and one control [an unrelated *C. perfringens* isolate]). WGS indicated all six epidemiologically linked isolates were highly related, with an average pairwise nucleotide difference of 3.35e-10 compared with an average pairwise nucleotide difference of 0.02 to the unrelated control isolate (*1*) (Supplementary Figure 1, https://stacks.cdc.gov/view/cdc/56097).

An investigation of the blood supplier and hospital X revealed no procedural deviations. The nontransfused plasma unit from the donor was quarantined. The donor was permanently deferred.

**California Cluster. **In August 2017, three apheresis platelet units and one unit of plasma were manufactured from an apheresis blood donation in California. One platelet unit was distributed to hospital Y, where it was divided into two aliquots, and two platelet units were distributed to hospital Z.

At hospital Y, one aliquot was transfused to a male who had received an autologous stem cell transplant (patient C); he developed vomiting, tachycardia, and hypotension approximately 15 minutes after transfusion initiation (Figure). Despite discontinuing transfusion, he died within 5 hours. Multiple posttransfusion blood cultures drawn after the transfusion reaction grew *K. pneumonia*e. Transfusion-transmitted bacterial infection was suspected, and Gram stain of platelet bag residuals was performed, revealing gram-negative rods. The blood supplier was immediately notified. *K. pneumoniae* was isolated from the platelet bag residuals.

Five hours before patient C’s transfusion, hospital Y’s second platelet aliquot had been transfused to a male (patient D) with myelodysplastic syndrome, fever, and neutropenia, who was on multiple broad-spectrum antibiotics. Approximately 9 hours after transfusion, the patient developed septic shock but recovered. Multiple posttransfusion blood cultures were negative, presumably a result of the antibiotic regimen.

When the blood supplier notified hospital Z of gram-negative rods identified in the residual aliquot transfused into patient C, the hospital returned a nontransfused platelet unit from which *K. pneumoniae* was later isolated. Hospital Z’s other platelet unit had been transfused 1 day before the notification. This platelet unit was transfused to a female (patient E) with disseminated intravascular coagulation and septic shock, for which she was receiving broad-spectrum antibiotics. She died the following day. Blood cultures obtained at the onset of sepsis (pretransfusion) and 8 hours after transfusion both grew multidrug-resistant *K. pneumoniae*.

The routine donor’s platelet bacterial screening collection, inoculated 24 hours after donation, was negative for growth through 5 days. The frozen plasma unit was not cultured and was discarded. *K. pneumoniae* isolates from three patient C blood cultures, patient C’s residual platelet product, and hospital Z’s nontransfused platelets had similar antibiograms and were highly related by WGS, differing by only two single nucleotide polymorphisms (Supplementary Figure 2, https://stacks.cdc.gov/view/cdc/56098). However, pretransfusion and posttransfusion *K. pneumoniae* isolates from patient E demonstrated multidrug resistance and were unrelated from the other isolates using WGS. Patient E’s possible source of sepsis was a pretransfusion urine infection with multidrug-resistant *K. pneumoniae*.

Investigation of the blood supplier and hospitals Y and Z indicated no procedural deviations. The donor met eligibility criteria and frequently donated platelets but had been deferred multiple times because of low hemoglobin. A platelet donation 9 months earlier was positive for *Enterobacter cloacae*. After the report of the *K. pneumoniae* cluster, medical history assessments did not identify donor bacterial infection risks. Nontransfused blood products from the implicated donation were quarantined, and the donor was permanently deferred.

## Discussion

Platelet-transmitted bacterial infections persist as a cause of transfusion-associated morbidity and mortality. Contamination of blood products most commonly occurs when skin microbiota are introduced during needle insertion but can also occur from asymptomatic donor bacteremia (*2*). Because the majority of platelets are stored at room temperature, bacteria can proliferate to clinically important levels by the time the unit is transfused (*3*). Approximately one in 5,000 platelet collections are contaminated with bacteria, and one in 100,000 platelet transfusions results in bacterial sepsis (*4*). Transfusion-transmitted bacterial infections are likely underdiagnosed (*2*) because recipients are often given broad spectrum antibiotics or have underlying medical conditions that increase sepsis risk, or the septic reaction might not be attributed to the transfusion.

Current practices to mitigate the risk for bacterial contamination of platelets include donor health screening, skin examination and disinfection, diversion of up to the first 40 mL of blood into a separate nontransfusable pouch to reduce the introduction of skin flora, visual inspection of platelet bags before transfusion, and aerobic bacterial culture screening (e.g., monitoring an aliquot for bacterial growth) at least 24 hours after platelet collection (*5*). Investigations confirmed that the Utah and California collection facilities followed current practices. This report highlights that, even when following current practices, the risk for fatalities persists, making additional, important interventions necessary.

The Food and Drug Administration (FDA) has several recommendations related to platelet contamination and donation.* FDA recommends that blood suppliers control the risk for bacterial contamination either by using a pathogen reduction device or performing bacterial detection at least once. Additional requirements when a pathogen is identified include product quarantine, organism identification, determination whether the pathogen is endogenous to the donor blood stream, and, if so, donor deferral.

Additional evidence-based risk mitigation strategies, including pathogen inactivation, rapid detection at point-of-use, and modification of screening bacterial culture protocols, can reduce the risk for platelet-transmitted bacterial sepsis (*3*). Implementation of these modified and alternative strategies in the United States has been supported by advice from the FDA’s Blood Products Advisory Committee but are not currently required (*3*). Pathogen inactivation technology was adopted in France, Belgium, and Switzerland, and although no confirmed septic transfusion reactions were reported from 2.3 million pathogen inactivation–treated platelet units, two possible cases have been reported after transfusion of pathogen inactivation–treated platelets (*6*). This same pathogen inactivation technology is approved by FDA for use with apheresis platelets and plasma in the United States.

Rapid bacterial detection devices, optimally used 72 hours after collection, can detect bacteria using <1 mL of platelet volume but only have detection limits of 10^3^–10^6^ organisms/mL. FDA has cleared one rapid device for extending platelet shelf life from 5 days to 7 days.^†^

Additional risk mitigation strategies modify existing bacterial culture screening protocols. Current methods differ by blood supplier, with most inoculating 8 mL into an aerobic blood culture microbial detection system sampled ≥24 hours after collection to allow for sufficient bacterial growth. If cultures are negative after 12–24 hours, platelet units are released and have a shelf life of up to 5 days, which can be extended up to 7 days with secondary testing (*3*). However, 8 mL of platelets sampled 24 hours after donation might not have sufficient bacterial loads to detect bacterial growth in the screening culture (*3*). Rather than using a fixed volume, one proposed strategy involves using a minimal proportional sample volume of 3.8% of the platelet total collection (*7*). In the United Kingdom, culture volumes of 16 mL are divided equally between aerobic and anaerobic culture bottles 36–48 hours after donation and have resulted in no recognized fatalities after approximately 1.8 million platelet units were transfused with shelf life extended to 7 days (*8*). However, on several reported occasions, platelet bags were suspected of contamination after visual inspection, and subsequent cultures confirmed contamination. In Ireland, repeat aerobic and anaerobic bacterial cultures are performed 4 days after collection to extend platelet shelf life to 7 days; no septic transfusion reactions have been reported after ˃100,000 apheresis collections (*3*). Although reporting by blood systems that have adopted modified culture screening methods is promising, demonstrating important clinical benefit is difficult because transfusion-associated bacterial sepsis is rare. However, when compared with current detection practices in the United States, methods based on larger volume culture, delayed sampling of platelets, and performing aerobic and anaerobic cultures after collection are likely to result in fewer cases of platelet-transmitted bacterial infections.

*C. perfringens*, a sporogenic gram-positive bacterium, has been rarely reported as the source of transfusion-associated sepsis (*4*). Disinfectants used for skin antisepsis during blood collection are not sporicidal and might be ineffective in removing *C. perfringens* from skin. *K. pneumoniae,* a gram-negative bacterium, is a common pathogen among transfusion-related fatalities (*9*). Both pathogens might not be inactivated by pathogen inactivation^§^ (*10*) but might have been detected with the modified culture strategies described above, which are not routinely practiced in the United States.

Blood collection services could consider implementing enhanced safety interventions to reduce further the risk for bacterial contamination of platelets. Clinicians could consider bacterial contamination when patients develop sepsis during or after a platelet transfusion and rapidly investigate these transfusion reactions.

**Summary**

**What is already known about this topic?**

Platelet-transmitted bacterial infections persist as a cause of transfusion-associated morbidity and mortality.

**What is added by this report?**

Whole genome sequencing was used to identify the source of fatal sepsis in three transfusion recipients resulting from bacterial contamination (*Clostridium perfringens* in Utah and *Klebsiella pneumoniae* in California) of platelet products.

**What are the implications for public health practice?**

Implementation of evidence-based strategies, including pathogen inactivation, rapid detection devices, and modified screening of bacterial culture protocols can mitigate the risk for bacterial contamination of platelets.

## Footnotes

* Control of Bacterial Contamination of Platelets, 21 C.F.R. Sect. 606.145 (2017). https://www.accessdata.fda.gov/scripts/cdrh/cfdocs/cfcfr/CFRSearch.cfm?fr=606.145; Donation suitability requirements, 21 C.F.R., Sect.630.30 (2017). https://www.accessdata.fda.gov/scripts/cdrh/cfdocs/cfcfr/CFRSearch.cfm?fr=630.30.

† https://www.fda.gov/downloads/BiologicsBloodVaccines/BloodBloodProducts/ApprovedProducts/SubstantiallyEquivalent510kDeviceInformation/UCM551535.pdf.

§ https://www.fda.gov/downloads/BiologicsBloodVaccines/BloodBloodProducts/ApprovedProducts/PremarketApprovalsPMAs/UCM427512.pdf.
